# Efficacy and safety of micafungin versus intravenous itraconazole as empirical antifungal therapy for febrile neutropenic patients with hematological malignancies: a randomized, controlled, prospective, multicenter study

**DOI:** 10.1007/s00277-015-2545-2

**Published:** 2015-11-24

**Authors:** Seong Hyun Jeong, Dae Young Kim, Jun Ho Jang, Yeung-Chul Mun, Chul Won Choi, Sung-Hyun Kim, Jin Seok Kim, Joon Seong Park

**Affiliations:** Department of Hematology-Oncology, Ajou University Hospital, Ajou Univesity School of Medicine, Suwon, South Korea; Department of Hematology, Asan Medical Center, University of Ulsan College of Medicine, Seoul, South Korea; Division of Hematology and Oncology, Samsung Medical Center, Sungkyunkwan University School of Medicine, Seoul, South Korea; Department of Internal Medicine, Ewha Womans University Mokdong Hospital, Ewha Womans Univerity School of Medicine, Seoul, South Korea; Division of Oncology-Hematology, Department of Internal Medicine, Korea University Guro Hospital, Seoul, South Korea; Department of Internal Medicine, Dong-A University College of Medicine, Busan, South Korea; Division of Hematology, Department of Internal Medicine, Severance Hospital, Yonsei University College of Medicine, Seoul, South Korea

**Keywords:** Micafungin, Empirical, Febrile neutropenia

## Abstract

Micafungin, a clinically important echinocandin antifungal drug, needs to be investigated as empirical therapy in febrile neutropenia in comparison with azole compounds. A prospective randomized study was conducted to compare clinical outcomes between micafungin and intravenous itraconazole as an empirical therapy for febrile neutropenia in hematological malignancies. The antifungal drug (micafungin 100 mg or itraconazole 200 mg IV once daily) was given for high fever that was sustained despite the administration of appropriate antibiotics. Treatment success was determined by composite end points based on breakthrough invasive fungal infection (IFI), survival, premature discontinuation, defervescence, and treatment of baseline fungal infection. Duration of fever, hospital stay, and overall survival (OS) were studied. A total of 153 patients were randomized to receive micafungin or itraconazole. The overall success rate was 7.1 % point higher in the micafungin group (64.4 vs. 57.3 %, *p* = 0.404), satisfying the statistical criteria for the non-inferiority of micafungin. The duration of fever and hospital stay were significantly shorter in the micafungin group (6 vs. 7 days, *p* = 0.014; 22 vs. 27 days, *p* = 0.033, respectively). Grade 3 adverse events including hyperbilirubinemia (2 vs. 7), elevation of transaminase levels (2 vs. 4), electrolyte imbalance (1 vs. 2), atrial fibrillation (1 vs. 0), and anaphylaxis (1 vs. 0) occurred in 7 and 13 patients in the micafungin (10.4 %) and itraconazole (18.8 %) groups, respectively. Micafungin, when compared with itraconazole, had favorably comparable success rate and toxicity profiles on febrile neutropenia in patients with hematological malignancies. In addition, it showed superior effect on shortening the hospital stay.

## Introduction

Febrile neutropenia (FN) is the most common and serious complication that can occur during intensive chemotherapy for patients with hematological malignancies [1, 2]. Since the incidence of fungal infection can reach 24 % among patients with leukemia and the mortality from invasive aspergillosis is as high as 50 %, proper empirical antifungal therapy is essential for patients with fever persisting despite initial broad-spectrum antibacterial therapy [3–5].

For empirical use, the antifungal drug should be less toxic than amphotericin B and more effective than the azole compounds against *Aspergillus* and some *Candida* species other than *C. albicans*. Echinocandins are considered one of the most adequate compounds for empirical therapy because of their wide antifungal coverage and high efficacy against *Aspergillus* and their excellent safety profile [6–8]. Micafungin is a novel echinocandin that has shown activity against the *Candida* and *Aspergillus* species by inhibiting the synthesis of 1,3-β-d-glucan, an essential component of the fungal cell wall [9]. Micafungin has shown its possible efficacy and safety as empirical therapy for febrile neutropenic patients in non-randomized studies [10–13]. In addition, micafungin may have an advantage over azole-resistant *Candida* and *Aspergillus* species [14, 15]. In Korea, itraconazole has been the only drug approved for empirical antifungal therapy since 2005 despite increasing demands for new antifungal empirical therapy. However, there has been no controlled study comparing other antifungal agents with itraconazole for empirical therapy among Koreans. Thus, in this study, we prospectively compared clinical outcomes of micafungin with intravenous itraconazole for empirical therapy in febrile neutropenia following anticancer chemotherapy for hematological disorders.

## Materials and methods

### Study design

This prospective randomized study was conducted from December 2012 through February 2014 at seven sites in South Korea. This study was designed as a non-inferiority test to document clinical outcomes of micafungin compared to those of itraconazole. The institutional review board of each participating institution approved this study design, and this study was conducted in accordance with the Declaration of Helsinki. Written informed consent was obtained from all patients before enrollment. The study protocol was registered with the National Institutes of Health clinical trial registry at www.clinicaltrials.gov (#NCT01344681).

Adult patients (age ≥ 18 years) were included if they had grade 4 neutropenia (absolute neutrophil count ≤500/μL) and high fever (≥38.4 °C at any time or ≥38.0 °C for 1 h by oral temperature) resulting from intensive anticancer chemotherapy for acute leukemia, highly aggressive lymphoma (Burkitt lymphoma, lymphoblastic lymphoma), or other hematological malignancies. To be eligible, patients had to have persistent high fever against proper broad-spectrum intravenous antibiotics for at least 72 h.

When patients met all the inclusion criteria, they were randomly assigned, in a 1:1 ratio by block randomization, to receive micafungin or itraconazole. Randomization was performed according to disease, patient age, sex, and risk stratification (remission induction or salvage therapy, high risk; consolidation, low risk). Subjects were automatically assigned by a pre-made random code with a Web-based electronic system. The randomized block design applied for the random code with risk stratification. Administration of oral antifungal agents for prophylaxis prior to the study was allowed.

Micafungin (Mycamine®, Astellas Pharma Tech Co., Ltd. Takaoka City, Japan) was administrated intravenously in normal saline at a dose of 100 mg over 60 min once daily. Itraconazole (Sporanox®, Janssen-Cilag Ltd., Buckinghamshire, England) was administrated intravenously at a dose of 200 mg twice daily for 2 days and then 200 mg once daily for 12 days. The minimum administration duration of the study drugs was 5 days. When the patients recovered from granulocytic nadir without fever, the empirical therapy was discontinued and treatment was considered to have been completed. Persistent high fever for 7 days after initiation of the antifungal drug was considered treatment failure, and a change in the assigned antifungal drug was allowed based on the individual decision of each investigator.

### Efficacy and safety

The primary end point was the overall success rate. Therapy was considered successful if the patient met all of the following criteria: (1) did not have a breakthrough invasive fungal infection (IFI); (2) survived for 7 days after therapy ended; (3) no premature discontinuation because of adverse events or lack of effects; (4) defervescence during granulocytic nadir; and (5) successful treatment of any baseline fungal infection. The breakthrough IFI was defined as proven or probable IFI with onset of symptoms of an IFI on day 3 or later after initiation of antifungal therapy following revised EORTC/MSG criteria [16]. These five components were used to determine the overall response rate [17].

The secondary end points were duration of fever, duration of febrile neutropenia, duration of hospital stay, and overall survival rate (OS). Toxicity profiles including physical findings and laboratory data were recorded and followed up until they were resolved using the National Cancer Institute Common Toxicity Criteria version 3.0.

### Statistical analysis

Analysis was based on an intention-to-treat approach from all patients who underwent randomization. The non-inferiority of micafungin in comparison with itraconazole was assessed and the inferiority margin was 10 %. The sample size was calculated from previous independent studies for empirical micafungin and itraconazole that had already shown response rates as 61.7 and 47 %, respectively [13, 18]. A sample size of 154 patients was achieved with the type 1 and type 2 errors of 0.025 and 0.8, respectively. The overall response rate of the antifungal agents was analyzed by Fisher’s exact test. Overall survival, duration of fever, and duration of hospital stay were assessed with the chi-square and log-rank tests. The two-sided *t* test was applied for patient characteristics, and a *p* value of less than 0.05 was considered to indicate statistical significance.

## Results

### Patient characteristics

**Fig. 1:**
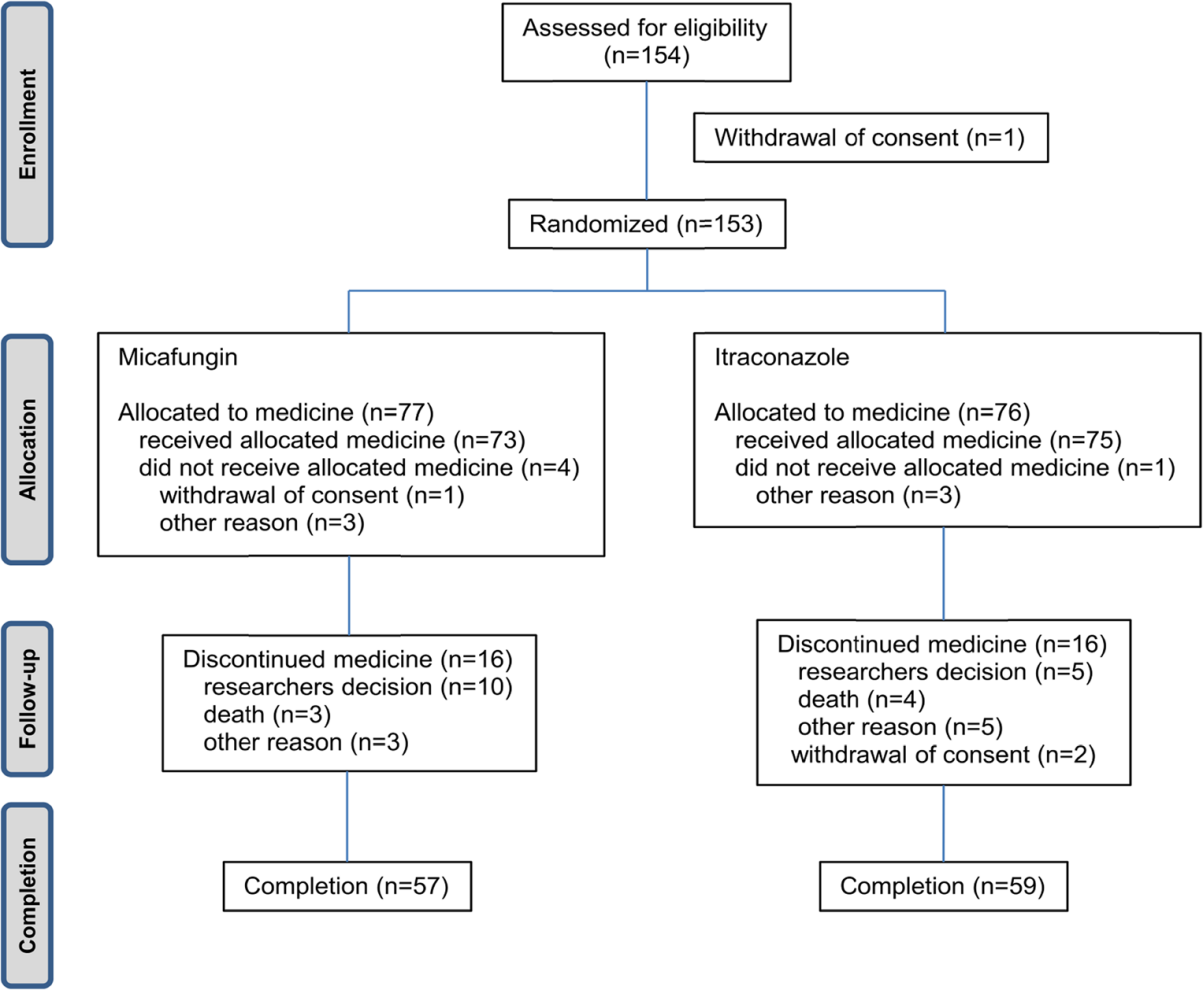
Flowchart of the study

A total of 153 patients were randomly assigned to the micafungin (*n* = 77) or itraconazole group (*n* = 76). Among them, 148 patients were treated with the study drugs (73 patients in the micafungin group and 75 patients in the itraconazole group). The completion rates of treatment with micafungin and itraconazole were 78.1 % (57/73) and 78.7 % (59/75), respectively (Fig. 1). All of the patients were native Koreans who received intensive anticancer chemotherapy for various hematological malignancies, mainly for acute leukemia. Sixty-three patients (86.3 %) were high-risk (remission induction or salvage) in the micafungin group and 64 patients (85.3 %) in the itraconazole group. Prophylactic antifungal therapy was used in 50.7 % of the patients in the micafungin group (fluconazole in 32 patients and itraconazole in 5) and 52.0 % in the itraconazole group (fluconazole in 34 patients and itraconazole in 5) before the initiation of the study drug. The median treatment duration of the study drug was 8 days in both groups. Patient characteristics including gender, age, diagnosis, and risk stratification were balanced (Table 1).

**Table 1:** Patient characteristics

		Micafungin group (*n* = 73)	Itraconazole group (*n* = 75)	*p* value
Gender, *n*	Male	42	40	0.624
	Female	31	35	
Age, years	Mean (range)	49 (20–85)	49 (20–78)	0.749
Diagnosis, *n* (%)	AML	45 (61.6)	43 (57.3)	0.926
	ALL	19 (26.0)	20 (26.6)	
	NHL	5 (6.8)	6 (8.0)	
	Burkitt’s	2	2	
	Lymphoblastic	3	4	
	Others	4 (5.4)	6 (8.0)	
	MDS	3	4	
	CMML	1	0	
	MM	0	2	
Underlying infection (other than fungal), *n* (%)	Total	30 (41.0)	22 (29.3)	0.169
	Bacterial	17	13	
	Viral	13	9	
Treatment setting, *n* (%)	Remission	44 (60.2)	41 (54.6)	0.629
	Salvage	17 (23.3)	23 (30.7)	
	Consolidation	12 (16.4)	11 (14.7)	
Antifungal prophylaxis, *n* (%)		37 (50.7)	39 (52.0)	0.869
Neutropenia duration, days	Median (range)	16 (4–63)	15 (5–26)	0.676
Treatment duration, days	Median (range)	8 (3–20)	8 (1–18)	0.452

*AML* acute myeloid leukemia, *ALL* acute lymphoblastic leukemia, *NHL* non-Hodgkin lymphoma, *MDS* myelodysplastic syndrome, *CMML* chronic myelomonocytic leukemia

### Success rate

The outcomes of the five composite parameters are shown in Table 2. There was no significant difference in each composite parameter between the two groups. A total of 16 patients had baseline fungal infection documented within 2 days from the initiation of study drugs (7 patients in the micafungin group, 9 patients in the itraconazole group). Four out of 7 patients (57.1 %) in the micafungin group and 5 out of 9 patients (55.6 %) in the itraconazole group have their documented fungal infection resolved with antifungal therapy during the study. Documented organisms and response are presented in Table 3. Breakthrough IFI occurred in 3 patients in the micafungin group with one proven IFI of *Candida* spp. and 2 probable cases. In the itraconazole group, there were two proven IFI of *Aspergillus* spp. and 3 probable cases. The overall success rates of micafungin and itraconazole were 64.4 and 57.3 %, respectively. The difference in the success rates (7.1 %) was not statistically significant (*p* = 0.404). Thus, these results satisfied non-inferiority criteria.

**Table 2:** Overall success rate

Parameter	Micafungin (%)	Itraconazole (%)	*p* value
Overall success rate	47/73 (64.4)	43/75 (57.2)	0.404
1. No breakthrough fungal infection^a^	70/73 (95.9)	70/75 (93.3)	0.719
2. Survived for at least 7 days after the end of therapy	67/73 (91.8)	67/75 (89.3)	0.780
3. No premature discontinuation for lack of efficacy or toxicity	50/73 (68.5)	49/75 (65.3)	0.729
4. Resolution of fever during neutropenia	49/73 (67.1)	45/75 (73.3)	0.473
5. Resolution of baseline fungal infection	4/7 (57.1)	5/9 (55.6)	1.000

^a^Breakthrough fungal infection: proven in 3 (*Aspergillus* spp. 2, *Candida* spp. 1) and probable in 5

**Table 3:** The treatment outcome of baseline fungal infection

Study group	Documented fungi	Resolution	Resolution rate
Micafungin	*Aspergillus flavus* *Candida albicans* *Candida tropicalis* *Candida* spp. *Trichosporon asahii*	1/1 2/2 1/2 0/1 0/1	57.1 %
Itraconazole	*Aspergillus* spp. *Candida albicans* *Candida tropicalis* *Candida* spp.	1/3 2/4 1/1 1/1	55.6 %

All of the fungi were documented within 2 days of the initiation of study drugs

### Defervescence, hospital stay, and overall survival

**Fig. 2:**
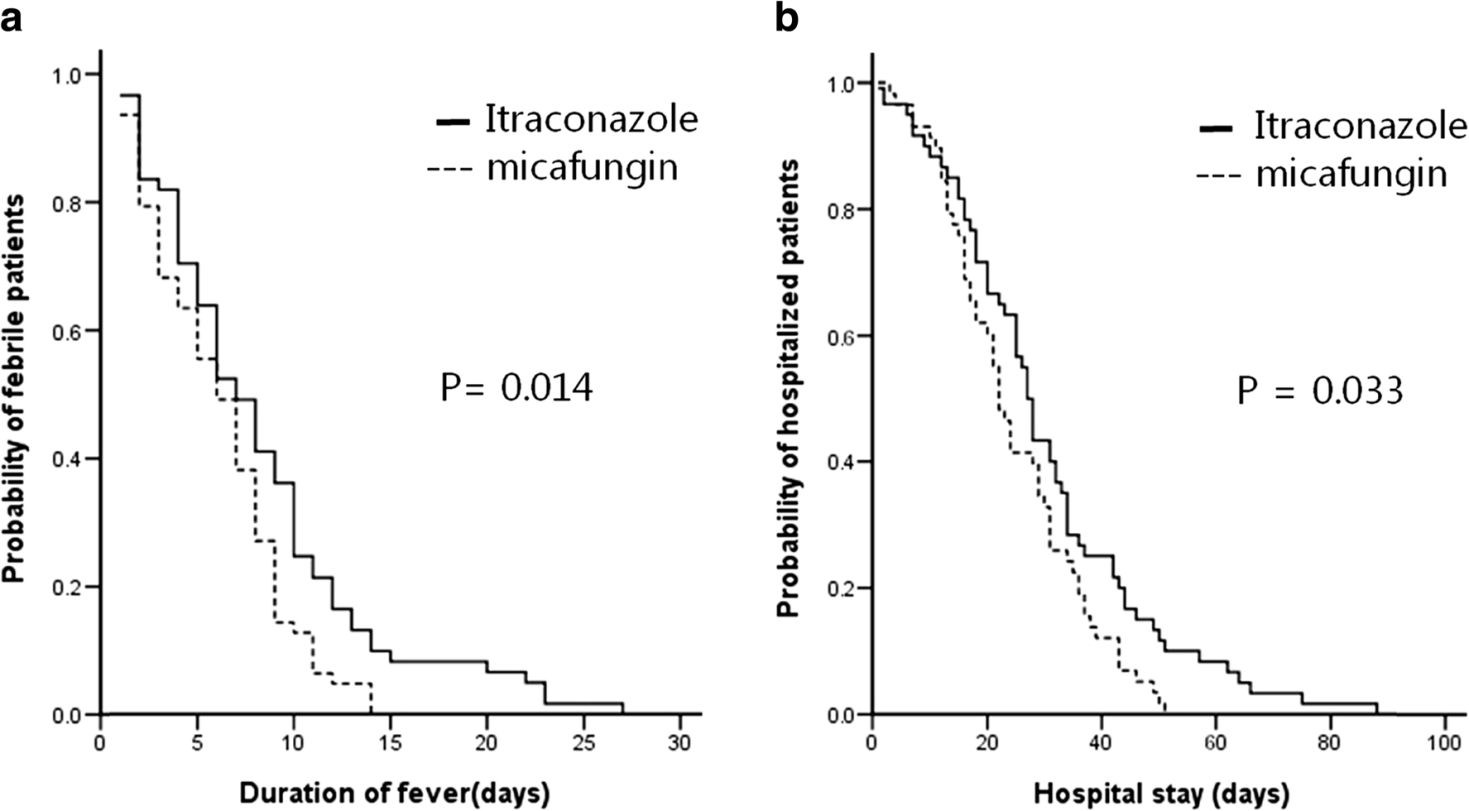
Duration of fever (**a**) and hospital stay (**b**)

The median duration of fever in the micafungin and itraconazole groups was 6.0 (95 % CI, 5–7) and 7.0 (95 % CI, 5–9) days, and the duration of fever was different between the two groups (*p* = 0.014) (Fig. 2a). The median duration of hospital stay in the micafungin group and itraconazole group was 22 (95 % CI, 19–25) and 27 (95 % CI, 25–29) days, and the duration of hospitalization was significantly lower in the micafungin group (*p* = 0.033) (Fig. 2b). The median OS in the micafungin group and itraconazole group was 12.77 (95 % CI, 8.92–16.62) and 9.27 (95 % CI, 5.27–13.27) months, respectively (*p* = NS). In responding patients, the median duration of drug delivery was 9.0 (95 % CI, 7–11) and 11.0 (95 % CI, 8–14) days in the micafungin and itraconazole group, respectively (*p* = NS).

### Adverse events

All 153 patients who were in the intention-to-treat population were included in the safety evaluation. The incidence and severity of adverse events were similar between the two groups (Table 4). Grade 3 hyperbilirubinemia, a well-known common adverse event of antifungal drugs, was observed in 2 and 7 patients in the micafungin and itraconazole group, respectively. Each case of atrial fibrillation and anaphylaxis was observed in the micafungin group, and the events were controlled with medications. Nine patients in the micafungin group (12.3 %) and 16 patients in the itraconazole group (21.3 %) discontinued study drugs prematurely because of adverse events. However, there was no adverse event-related death in both groups and 3 and 4 patients died of fatal infections in the micafungin and itraconazole group, respectively.

**Table 4:** Adverse events

Adverse events	Micafungin (*n* = 73)		Itraconazole (*n* = 75)	
	All grades (*n*)	Grade III–IV (*n*)	All grades (*n*)	Grade III–IV (*n*)
Hyperbilirubinemia Elevation of transaminase level Electrolyte imbalance Atrial fibrillation Anaphylaxis Delirium Insomnia Skin rash	10 5 18 1 1 3 4 9	2 2 1 1 1 0 0 0	11 19 16 0 0 2 1 5	7 4 2 0 0 0 0 0
Total	51	11	44	17

Probability of grade 3 or more severe adverse events was not different between the two groups. Each case of atrial fibrillation and anaphylaxis occurred in the micafungin group, which should be reminded prudently despite the fact that the events were well controlled with medications

## Discussion

This randomized, multicenter trial has shown that empirical micafungin is as effective as itraconazole for patients with febrile neutropenia. Micafungin compares favorably with itraconazole in terms of overall success rates and toxicity profiles. Theoretically, the echinocandins have advantages over the azole or polyene compounds as empirical therapy because of their wide coverage and high efficacy against invasive aspergillosis [7, 8]. The efficacy and safety of the echinocandins as empirical antifungal therapy were demonstrated with caspofungin by Walsh et al. [17]. When compared to liposomal amphotericin B, caspofungin showed favorable efficacy and a better toxicity profile and tolerance. Micafungin is another new echinocandin that has considerable efficacy and a notable toxicity profile [11, 12, 19, 20]. We previously investigated the possible efficacy and safety of micafungin as an empirical therapy in febrile neutropenia with a small observational study [13]. However, there has been no randomized study to compare micafungin with other agents as empirical therapy. As far as we know, this is the first randomized study of micafungin as empirical therapy in febrile neutropenia. Since itraconazole has been the only drug allowed for empirical therapy in Korea since 2005, we had to compare micafungin with itraconazole in this study.

In this study, the overall success rate evaluated with five composite scores was somewhat higher (64.4 % for the micafungin group and 57.2 % for the itraconazole group) than that of previous studies on empirical therapy with liposomal amphotericin, voriconazole, and caspofungin [17, 21, 22]. In this study, the patients started empirical antifungal drugs earlier than those in previous studies when they had sustained fever more than 72 h despite proper antibiotics therapy, whereas 96–120 h in other studies. Another possible explanation is that the patients undergoing hematopoietic stem cell transplantation (HSCT) were not included in this study unlike other empirical trials, since micafungin was allowed to be used as prophylactic therapy for HSCT in Korea.

Breakthrough fungal infection occurred in 3 patients (4 %) in the micafungin group. One patient was proven to have systemic *Candida* infection. Although micafungin has potent activity against *Candida* spp. and *Aspergillus* spp., recent research has shown that breakthrough IFI occurred most commonly due to *Candida* spp. for high minimum inhibitory concentration and/or relative insensitivity to echinocandins [23]. Thus, systemic candidemia should be considered when the patients show clinical aggravation despite micafungin treatment.

Another strength of this study is that the study population consists of homogeneous patients undergoing intensive chemotherapy for hematological malignancies where anticipated mortality from IFIs is the highest. Although some studies pursued a preemptive strategy with recent advances of sensitive mycological tests and imaging techniques [24, 25], the most effective strategy in preventing or treating invasive fungal disease in febrile neutropenia is still under debate. Since the mortality from IFIs is considerable in patients with acute leukemia suffering from febrile neutropenia, especially while undergoing induction chemotherapy, empirical therapy is still widely accepted in this clinical setting [26]. This study showed the efficacy of micafungin as an empirical therapy option in this high-risk population.

Duration of hospital stay was adopted as an indication of the overall effectiveness of empirical antifungal treatment. It could be a good surrogate for the combined results of treatment success, adverse effects, and other clinical aspects. The duration of neutropenia was not different between the two groups, and duration of fever was only modestly shorter in the micafungin group. However, the duration of hospitalization was shorter in the micafungin group by median 5 days (22 vs. 27 days, *p* = 0.033). This may benefit patients because it allows treatment to progress to the next step in a timely manner and dose intensity can be maintained throughout the entire treatment while treatment costs can also be decreased. Although not proven in this study, this may be translated to a better long-term treatment outcome. Considering the shorter hospital stay, micafungin seems to have advantages over itraconazole despite no significant difference in the overall success rate by a composite score between the two groups.

In summary, micafungin was effective as empirical therapy in sustained fever and neutropenia with acceptable toxicity profiles in comparison with itraconazole. Additionally, it significantly decreased the duration of hospitalization when compared to itraconazole. Among available antifungal agents, micafungin could be a favorable option for empirical antifungal treatment in febrile neutropenia.

## References

[1] Hamalainen S, Kuittinen T, Matinlauri I, Nousiainen T, Koivula I, Jantunen E (2008). Neutropenic fever and severe sepsis in adult acute myeloid leukemia (AML) patients receiving intensive chemotherapy: causes and consequences. Leuk Lymphoma.

[2] Jagarlamudi R, Kumar L, Kochupillai V, Kapil A, Banerjee U, Thulkar S (2000). Infections in acute leukemia: an analysis of 240 febrile episodes. Med Oncol.

[3] Pagano L, Caira M, Candoni A, Offidani M, Martino B, Specchia G, Pastore D, Stanzani M, Cattaneo C, Fanci R, Caramatti C, Rossini F, Luppi M, Potenza L, Ferrara F, Mitra ME, Fadda RM, Invernizzi R, Aloisi T, Picardi M, Bonini A, Vacca A, Chierichini A, Melillo L, de Waure C, Fianchi L, Riva M, Leone G, Aversa F, Nosari A (2010). Invasive aspergillosis in patients with acute myeloid leukemia: a SEIFEM-2008 registry study. Haematologica.

[4] Rotstein C, Bow EJ, Laverdiere M, Ioannou S, Carr D, Moghaddam N (1999). Randomized placebo-controlled trial of fluconazole prophylaxis for neutropenic cancer patients: benefit based on purpose and intensity of cytotoxic therapy. The Canadian Fluconazole Prophylaxis Study Group. Clin Infect Dis.

[5] Viscoli C, Girmenia C, Marinus A, Collette L, Martino P, Vandercam B, Doyen C, Lebeau B, Spence D, Krcmery V, De Pauw B, Meunier F (1999). Candidemia in cancer patients: a prospective, multicenter surveillance study by the Invasive Fungal Infection Group (IFIG) of the European Organization for Research and Treatment of Cancer (EORTC). Clin Infect Dis.

[6] Denning DW (2003). Echinocandin antifungal drugs. Lancet.

[7] Bartizal K, Gill CJ, Abruzzo GK, Flattery AM, Kong L, Scott PM, Smith JG, Leighton CE, Bouffard A, Dropinski JF, Balkovec J (1997). In vitro preclinical evaluation studies with the echinocandin antifungal MK-0991 (L-743,872). Antimicrob Agents Chemother.

[8] van Burik JA, Ratanatharathorn V, Stepan DE, Miller CB, Lipton JH, Vesole DH, Bunin N, Wall DA, Hiemenz JW, Satoi Y, Lee JM, Walsh TJ, National Institute of A, Infectious Diseases Mycoses Study G (2004). Micafungin versus fluconazole for prophylaxis against invasive fungal infections during neutropenia in patients undergoing hematopoietic stem cell transplantation. Clin Infect Dis.

[9] Pettengell K, Mynhardt J, Kluyts T, Lau W, Facklam D, Buell D, Group FKSAS (2004). Successful treatment of oesophageal candidiasis by micafungin: a novel systemic antifungal agent. Aliment Pharmacol Ther.

[10] Goto N, Hara T, Tsurumi H, Ogawa K, Kitagawa J, Kanemura N, Kasahara S, Yamada T, Shimizu M, Nakamura M, Matsuura K, Moriwaki H (2010). Efficacy and safety of micafungin for treating febrile neutropenia in hematological malignancies. Am J Hematol.

[11] Yoshida M, Tamura K, Imamura M, Niitsu Y, Sasaki T, Urabe A, Ohyashiki K, Naoe T, Kanamaru A, Tanimoto M, Masaoka T (2012). Efficacy and safety of micafungin as an empirical antifungal therapy for suspected fungal infection in neutropenic patients with hematological disorders. Ann Hematol.

[12] Yanada M, Kiyoi H, Murata M, Suzuki M, Iwai M, Yokozawa T, Baba H, Emi N, Naoe T (2006). Micafungin, a novel antifungal agent, as empirical therapy in acute leukemia patients with febrile neutropenia. Intern Med.

[13] Park JS, Kim DH, Choi CW, Jeong SH, Choi JH, Kim K, Kim SJ, Jung CW, Yang DH, Jang JH (2010). Efficacy and safety of micafungin as an empirical antifungal agent for febrile neutropenic patients with hematological diseases. Acta Haematol.

[14] Richards TS, Oliver BG, White TC (2008). Micafungin activity against Candida albicans with diverse azole resistance phenotypes. J Antimicrob Chemother.

[15] Warn PA, Morrissey G, Morrissey J, Denning DW (2003). Activity of micafungin (FK463) against an itraconazole-resistant strain of Aspergillus fumigatus and a strain of Aspergillus terreus demonstrating in vivo resistance to amphotericin B. J Antimicrob Chemother.

[16] De Pauw B, Walsh TJ, Donnelly JP, Stevens DA, Edwards JE, Calandra T, Pappas PG, Maertens J, Lortholary O, Kauffman CA, Denning DW, Patterson TF, Maschmeyer G, Bille J, Dismukes WE, Herbrecht R, Hope WW, Kibbler CC, Kullberg BJ, Marr KA, Munoz P, Odds FC, Perfect JR, Restrepo A, Ruhnke M, Segal BH, Sobel JD, Sorrell TC, Viscoli C, Wingard JR, Zaoutis T, Bennett JE, European Organization for R, Treatment of Cancer/Invasive Fungal Infections Cooperative G, National Institute of A, Infectious Diseases Mycoses Study Group Consensus G (2008). Revised definitions of invasive fungal disease from the European Organization for Research and Treatment of Cancer/Invasive Fungal Infections Cooperative Group and the National Institute of Allergy and Infectious Diseases Mycoses Study Group (EORTC/MSG) Consensus Group. Clin Infect Dis.

[17] Walsh TJ, Teppler H, Donowitz GR, Maertens JA, Baden LR, Dmoszynska A, Cornely OA, Bourque MR, Lupinacci RJ, Sable CA, dePauw BE (2004). Caspofungin versus liposomal amphotericin B for empirical antifungal therapy in patients with persistent fever and neutropenia. N Engl J Med.

[18] Boogaerts M, Winston DJ, Bow EJ, Garber G, Reboli AC, Schwarer AP, Novitzky N, Boehme A, Chwetzoff E, De Beule K, Itraconazole Neutropenia Study G (2001). Intravenous and oral itraconazole versus intravenous amphotericin B deoxycholate as empirical antifungal therapy for persistent fever in neutropenic patients with cancer who are receiving broad-spectrum antibacterial therapy. A randomized, controlled trial. Ann Intern Med.

[19] Cornely OA, Marty FM, Stucker F, Pappas PG, Ullmann AJ (2011). Efficacy and safety of micafungin for treatment of serious Candida infections in patients with or without malignant disease. Mycoses.

[20] Kuse ER, Chetchotisakd P, da Cunha CA, Ruhnke M, Barrios C, Raghunadharao D, Sekhon JS, Freire A, Ramasubramanian V, Demeyer I, Nucci M, Leelarasamee A, Jacobs F, Decruyenaere J, Pittet D, Ullmann AJ, Ostrosky-Zeichner L, Lortholary O, Koblinger S, Diekmann-Berndt H, Cornely OA, Micafungin Invasive Candidiasis Working G (2007). Micafungin versus liposomal amphotericin B for candidaemia and invasive candidosis: a phase III randomised double-blind trial. Lancet.

[21] Walsh TJ, Pappas P, Winston DJ, Lazarus HM, Petersen F, Raffalli J, Yanovich S, Stiff P, Greenberg R, Donowitz G, Schuster M, Reboli A, Wingard J, Arndt C, Reinhardt J, Hadley S, Finberg R, Laverdiere M, Perfect J, Garber G, Fioritoni G, Anaissie E, Lee J, National Institute of A, Infectious Diseases Mycoses Study G (2002). Voriconazole compared with liposomal amphotericin B for empirical antifungal therapy in patients with neutropenia and persistent fever. N Engl J Med.

[22] Walsh TJ, Finberg RW, Arndt C, Hiemenz J, Schwartz C, Bodensteiner D, Pappas P, Seibel N, Greenberg RN, Dummer S, Schuster M, Holcenberg JS (1999). Liposomal amphotericin B for empirical therapy in patients with persistent fever and neutropenia. National Institute of Allergy and Infectious Diseases Mycoses Study Group. N Engl J Med.

[23] Chan TS, Gill H, Hwang YY, Sim J, Tse AC, Loong F, Khong PL, Tse E, Leung AY, Chim CS, Lie AK, Kwong YL (2014). Breakthrough invasive fungal diseases during echinocandin treatment in high-risk hospitalized hematologic patients. Ann Hematol.

[24] Aguilar-Guisado M, Martin-Pena A, Espigado I, Ruiz Perez de Pipaon M, Falantes J, de la Cruz F, Cisneros JM (2012). Universal antifungal therapy is not needed in persistent febrile neutropenia: a tailored diagnostic and therapeutic approach. Haematologica.

[25] Maschmeyer G, Heinz WJ, Hertenstein B, Horst HA, Requadt C, Wagner T, Cornely OA, Loffler J, Ruhnke M, Investigators Is (2013). Immediate versus deferred empirical antifungal (IDEA) therapy in high-risk patients with febrile neutropenia: a randomized, double-blind, placebo-controlled, multicenter study. Eur J Clin Microbiol Infect Dis.

[26] Cordonnier C, Pautas C, Maury S, Vekhoff A, Farhat H, Suarez F, Dhedin N, Isnard F, Ades L, Kuhnowski F, Foulet F, Kuentz M, Maison P, Bretagne S, Schwarzinger M (2009). Empirical versus preemptive antifungal therapy for high-risk, febrile, neutropenic patients: a randomized, controlled trial. Clin Infect Dis.

